# Change in dietary inflammatory index score is associated with control of long-term rheumatoid arthritis disease activity in a Japanese cohort: the TOMORROW study

**DOI:** 10.1186/s13075-021-02478-y

**Published:** 2021-04-08

**Authors:** Yoshinari Matsumoto, Nitin Shivappa, Yuko Sugioka, Masahiro Tada, Tadashi Okano, Kenji Mamoto, Kentaro Inui, Daiki Habu, James R. Hebert, Tatsuya Koike

**Affiliations:** 1 Shirahama Foundation for Health and Welfare, Search Institute for Bone and Arthritis Disease (SINBAD), Nishimuro-gun, Shirahama-cho 1447, Wakayama, 649-2211 Japan; 2 Department of Medical Nutrition, Osaka City University Graduate School of Human Life Science, Sumiyoshi-ku, Sugimoto-cho 3-3-138, Osaka, 558-8585 Japan; 3 Cancer Prevention and Control Program and Department of Epidemiology and Biostatistics, Arnold School of Public Health, University of South Carolina, 915 Greene Street, Suite 241-2, Columbia, SC 29208 USA; 4 Department of Nutrition, Connecting Health Innovations LLC, 1417 Gregg St., Columbia, SC 29201 USA; 5 Center for Senile Degenerative Disorders (CSDD), Osaka City University Medical School, Abeno-ku, Asahimachi 1-4-3, Osaka, 545-8585 Japan; 6 Departments of Orthopaedic Surgery, Osaka City University Medical School, Abeno-ku, Asahimachi 1-4-3, Osaka, 545-8585 Japan

**Keywords:** Inflammation, Nutrition, Diet, Lifestyle, Rheumatoid arthritis

## Abstract

**Background:**

The dietary inflammatory index (DII®), a quantitative measure of the inflammatory potential of daily food and nutrient intake, and associations between a variety of health outcomes have been reported. However, the association between DII score and disease activity of rheumatoid arthritis (RA) is unclear. Therefore, this study was designed to test whether higher DII score contributes to disease activity and as a corollary, whether reducing DII score helps to achieve or maintain low disease activity or remission in patients with RA.

**Methods:**

We performed a cross-sectional and longitudinal analysis using 6 years of data (from 2011 to 2017) in TOMORROW, a cohort study consisting of 208 RA patients and 205 gender- and age-matched controls started in 2010. Disease activity of RA patients was assessed annually using DAS28-ESR (disease activity score 28 joints and the erythrocyte sedimentation rate) as a composite measure based on arthritic symptoms in 28 joints plus global health assessment and ESR. Dietary data were collected in 2011 and 2017 using the brief-type self-administered diet history questionnaire (BDHQ). Energy-adjusted DII (E-DII™) score was calculated using 26 nutrients derived from the BDHQ. Data were analyzed with two-group comparisons, correlation analysis, and multivariable logistic regression analysis.

**Results:**

One hundred and seventy-seven RA patients and 183 controls, for whom clinical and dietary survey data were available, were analyzed. RA patients had significantly higher E-DII (pro-inflammatory) score compared to controls both in 2011 and 2017 (*p* < 0.05). In RA patients, E-DII score was not a factor associated with significant change in disease activity. However, anti-inflammatory change in E-DII score was associated maintaining low disease activity (DAS28-ESR ≤ 3.2) or less for 6 years (OR 3.46, 95% CI 0.33–8.98, *p* = 0.011).

**Conclusions:**

The diets of RA patients had a higher inflammatory potential than controls. Although E-DII score was not a factor associated with significant disease activity change, anti-inflammatory change in E-DII score appeared to be associated with maintaining low disease activity in patients with RA.

**Trial registration:**

UMIN Clinical Trials Registry, UMIN000003876. Registered 7 Aug 2010—retrospectively registered.

## Background

Rheumatoid arthritis (RA) is an autoimmune disease of unknown exact cause, defined by chronic inflammation. RA primarily results in destruction of joints and bones caused by chronic inflammation. RA is triggered locally and systemically and by the over-activation of immune cells and joint synovial cells. Reducing inflammation is one of the key strategies for treating RA [1]. RA is mainly treated by pharmacotherapy, which includes disease-modifying antirheumatic drugs (DMARDs), glucocorticoid, and biological or targeted synthetic DMARDs (b-, tsDMARDs) which are molecularly targeted drugs suppressing the immune mediators or JAK-STAT (Janus activated kinase-signal transducers and activators of transcription) pathway, a downstream signaling pathway in cytokine-bound cells. Suppression of various points of inflammation pathways is important for controlling disease activity of RA [2].

The anti-inflammatory function of some nutrients has been reported in a variety of basic and clinical investigations and interventional studies, and many studies in patients with RA have been reported [3]. Classically, it has been reported that the intake of anti-inflammatory fatty acids such as n-3 fatty acids and γ-linolenic acid in RA patients reduces inflammation and disease activity [4]. We reported that intake of monounsaturated fatty acids may be associated with suppression of disease activity in patients with RA [5]. In addition, there is a report that the intake of micro-nutrients such as curcumin (present in turmeric), which has anti-inflammatory effects, can reduce disease activity in RA patients [6]. Therefore, dietary therapy focusing on the function of nutrients in RA patients is attracting attention [7]. However, it is also true that there are very few studies reporting findings that have used evidence-based methods for assessing exposure. Dietary and nutritional therapies to suppress inflammation in RA patients include therapies that focus only on single nutrients, and dietary patterns reflecting differences in combinations of foods and nutrients [8]. In dietary-pattern interventions, it has been reported that a Mediterranean diet pattern that focuses on increasing consumption of plant foods and reducing intake of animal products has a beneficial effect on disease activity [9, 10]. Likewise, an anti-inflammatory dietary pattern that is rich in anti-inflammatory nutrients including n-3 fatty acids, antioxidant nutrients such as vitamins C and E, and fiber and probiotics that can improve intestinal flora also has a beneficial effect on disease activity [11].

Though there are several tools to evaluate dietary patterns, the dietary inflammatory index (DII®) focuses specifically on the effects of food and nutrients on inflammation [12]. The DII is a literature-derived index calculated from various food and nutrients that have been shown to have an effect on inflammation, and associations between the DII and a variety of outcomes have been reported, including risk of developing and dying of heart disease [13], colorectal cancer incidence [14], and overall cancer mortality [15]. The DII score is unique in being designed specifically to quantify the degree of inflammation from dietary content; there is no other formulation aimed specifically at describing diet-associated inflammation that has been used so widely. However, the association between DII and disease activity of RA is unclear. We investigate whether higher inflammatory index might contribute to disease activity, and reducing inflammatory diet components might help achieve or maintain low disease activity or remission in patients with RA. This study analyzed data within a prospective cohort study with the aim of comparing DII scores between RA patients and gender- and age-matched controls and examining the association of DII with RA disease activity.

## Methods

### Study population

**Fig. 1:**
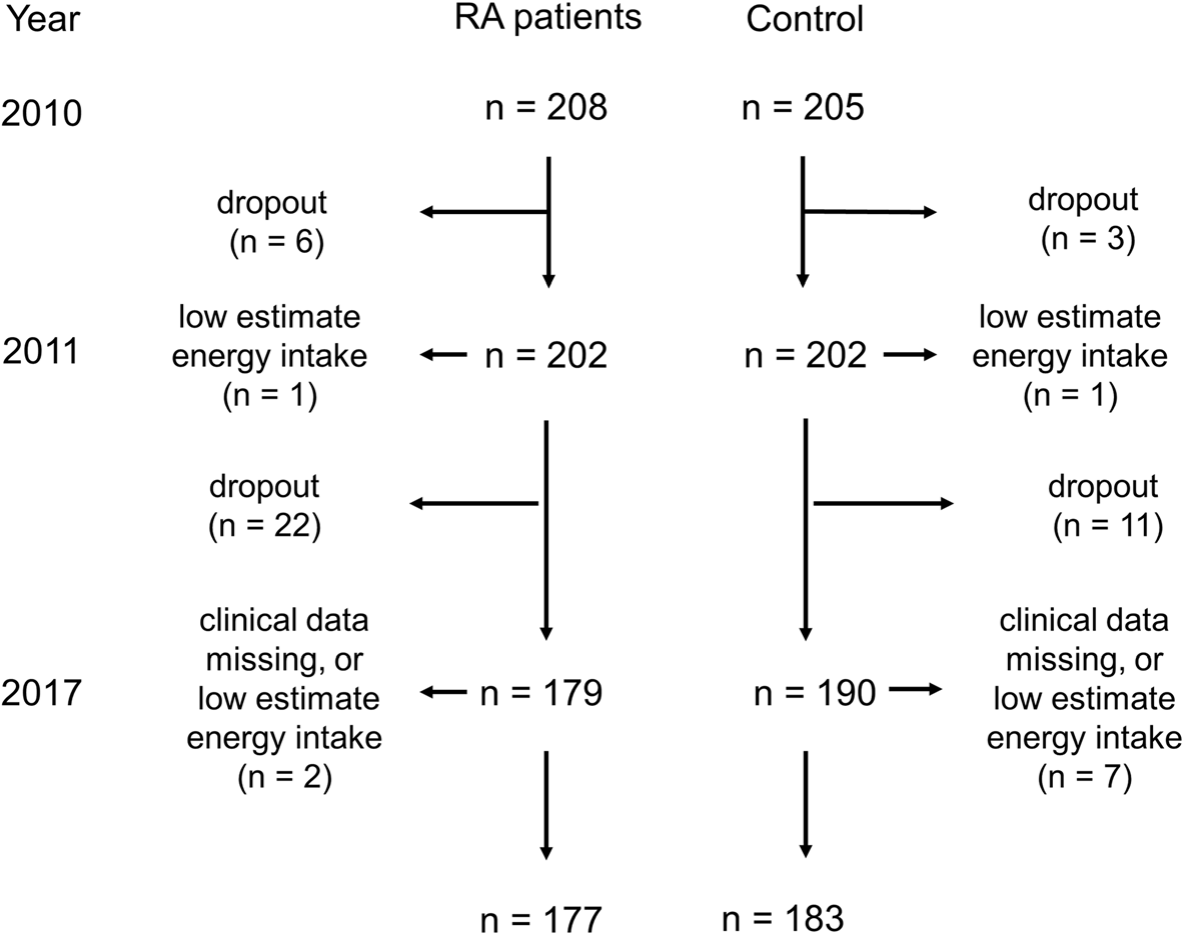
The flow chart of study subjects. In this study, we analyzed the data of 177 RA patients and 183 controls. RA, rheumatoid arthritis

In this study, we used data from the TOMORROW (Total management of risk factors in rheumatoid arthritis patients to lower morbidity and mortality) study, a 10-year prospective cohort study consisting of 208 RA patients and 205 gender- and age-matched non-RA controls that began in 2010 at Osaka City University Hospital in Osaka, Japan [5]. The study concept and details of the design, including recruitment methods of the control subjects, were previously reported [5, 16, 17]. Briefly, patients were recruited so that approximately half of the patients would be bDMARD users to investigate the effect of bDMARDs on a variety of outcomes, and disease activity was assessed in annual surveys. In this study, we used 6 years of data from 2011 to 2017 because we administered nutrition surveys in two of these years (i.e., 2011 and 2017). After excluding subjects who had died, who wished to withdraw consent, or for whom relocation made it difficult to continue the study, missing some clinical data, poorly validated nutrient intake survey results, there were 177 RA patients and 183 controls in 2017 (Fig. 1).

This study was conducted after written informed consent was obtained from all study subjects in accordance with the Declaration of Helsinki, and the research protocol was approved by the Ethics Committee of the Osaka City University Hospital (approval number 1660). This study was registered as a clinical observation one in University Hospital Medical Information Network, UMIN000003876.

### Subject’s basic characteristics

Anthropometric measurements were collected as previously reported [5]. Smoking and drinking history was based on 2010 data, i.e., at the time of enrollment, and medication for RA was surveyed annually.

### Food and nutrients intake status

Daily dietary and nutrient intake status were assessed in 2011 and 2017 using the brief-type self-administered diet history questionnaire (BDHQ), a validated instrument which queries the intake of 60 foods from which about 100 nutrients are estimable [18, 19]. As previously reported, a subject whose intake was less than 600 kcal/day (deemed to be a gross underestimate) was excluded from the analysis to maintain the validity of the dietary survey [5]. Therefore, data from 177 RA patients and 183 control subjects were finally included in the analysis.

### Clinical assessment

Disease activity was annually assessed in 2011 and 2017 for the TOMORROW study using the DAS28-ESR (Disease activity scores in 28 joints using erythrocyte sedimentation rates), which is a composite score derived from four components to evaluate the disease activity of RA [20]. The four components are (1) the number of swollen joints out of the 28 joints assessed, (2) the number of tender joints out of the 28, (3) ESR as a marker of systemic inflammation, and (4) global assessment of health indicated by a visual analog scale. Disease activity was classified according to the European League against Rheumatism (EULAR) disease activity classification, with DAS28-ESR 3.2 or less as low disease activity (LDA) [21]. Thirty-five patients with a DAS28-ESR of 3.2 or less on all assessments across study years during the 6-year period from 2011 to 2017 were assigned to be in LDA group. Activity of daily living (ADL) was assessed using the modified health assessment questionnaire disability index (mHAQ-DI) [22]. The degree of change in disease activity between 2011 and 2017 was assessed based on the EULAR response [23]. Specifically, a DAS of less than 5.1 in 2011 and improved 0.6 or more in 2017 was evaluated as a responder, and a DAS of 5.1 or higher in 2011 and improved 1.2 or more in 2017 was also evaluated as a responder.

### Calculation of DII/E-DII

DII score was calculated based on the BDHQ data. The DII may be calculated from the intake of up to 45 food parameters (i.e., foods and nutrients) [12]. However, only 26 items (alcohol, thiamine, riboflavin, vitamin B6, vitamin B12, beta carotene, carbohydrate, cholesterol, energy, fat, fiber, folic acid, iron, magnesium, monounsaturated fatty acids, niacin, n-3 polyunsaturated fatty acids, PUFA (polyunsaturated fatty acid), n-6 PUFA, protein, total PUFA, saturated fatty acids, vitamin A, vitamin C, vitamin D, vitamin E, zinc) were available from the BDHQ and were used for calculating the DII. The DII score is a standardized score, based on *z*-scores and conversion to proportions that are centered on zero as the reference point. The scoring system was configured with more anti-inflammatory scores being negative and more pro-inflammatory scores being positive [12, 24]. Because total energy intake affects overall nutrient intake, energy-adjusted DII (E-DII™) scores were calculated using the density approach, and these were used in this study analyses. Calculation of the E-DII, which is similar in every other way to the DII, required employing a world comparative database also adjusted for energy intake [25]. All analyses reported here are based on the E-DII. The change value in E-DII from 2011 to 2017 was presented as ΔE-DII calculated as follows: ΔE-DII = E-DII in 2017 − E-DII in 2011.

### Statistical analysis

The Statistical Package for Social Sciences (SPSS) software version 25.0 (IBM Corp, Armonk, NY, USA) was used for all statistical analyses. The normality of the data was assessed with the Shapiro-Wilk test. Data comparison between the two groups was performed by Mann-Whitney *U* test or Wilcoxon signed-rank test. The statistical differences in the categorical data were tested by Fisher’s exact test. The effect size was calculated as *r* for numeric data and Cramer’s *V* for categorical data. Spearman’s rank-order correlation coefficient was calculated as correlation coefficient. The covariates used in multivariable logistic regression analysis were examined using the forced-entry method. In the cross-sectional analysis of the association between disease activity and E-DII score in RA patients, we included gender, age (cut-off value was overall median age; years < 61 or years ≥ 61), body mass index (BMI) by group (18.5 kg/m^2^ ≤ BMI < 25 kg/m^2^; normal range, or other), smoking (with or without smoking within 1 year), anti-cyclic citrullinated peptide (CCP) antibody (positive or negative), and E-DII score (positive or negative—no subject had a score of zero). Because the E-DII score is based on *z*-scores that are converted to proportions and centered on zero, values can take on either negative or positive values. The lower the score, the more anti-inflammatory the diet. The use of bDMARDs in each year was included because, the medications for RA, bDMARDs are the ones that are most likely to affect disease activity, based on the algorithms for medication in RA patients [2].

In the longitudinal analysis, we tried to identify the factors to keep LDA or less during 6 years. Because 35 patients were able to maintain their disease severity at LDA or less during the 6-year interval, to ensure that the event rate was greater than 5 to guarantee a confidence interval [26], the number of items entered was set to 7. We also calculated bootstrap confidence intervals (repeated 1000 times). We included in the same items that put in 2011 cross-sectional logistic regression analysis. For E-DII, the item was shown to have decreased in 2017 compared to 2011 (ΔE-DII score negative). Since it has been reported that alcohol consumption may affect disease activity [27], we also investigated the effect of alcohol consumption instead of E-DII as an item. *P* values less than 0.05 (2-tailed) were considered statistically significant.

## Results

### Comparison of E-DII score in RA patients and controls

**Fig. 2:**
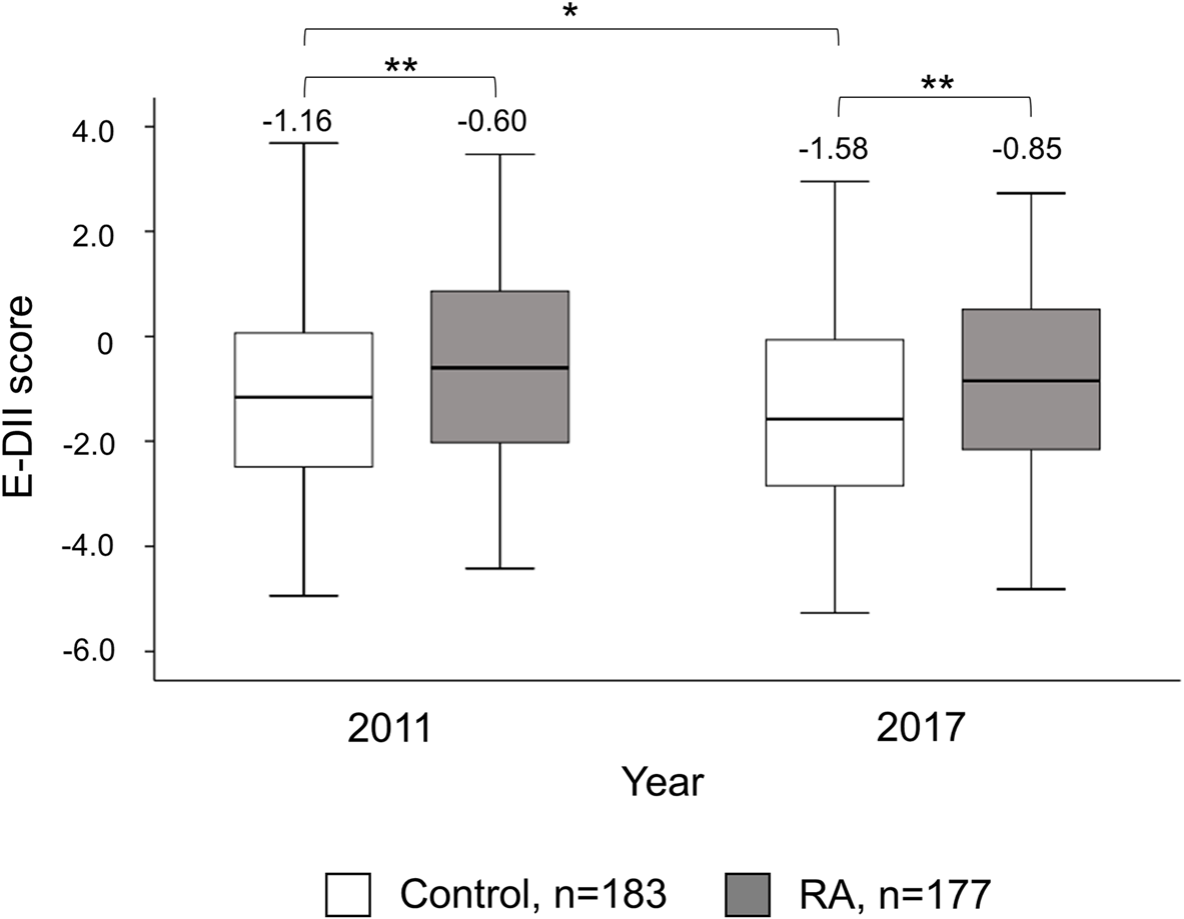
E-DII in control and RA patients. Comparison of E-DII between controls (white, *n* = 183) and RA patients (grayscale, *n* = 177) in 2011 and 2017. The data are presented as boxplots. **p < 0.01, *p < 0.05. Effect size for each statistic; control vs RA: in 2011 = 0.14, in 2017 = 0.16. 2011 vs 2017: control = 0.16, RA = 0.12. The figures shown above the boxplots are the median values

The basic demographics of the control group and RA patients are shown in Table 1. Smoking was significantly more common in RA patients. Regarding E-DII score, RA patients had significantly higher E-DII score (pro-inflammatory diet) both in 2011 and 2017 compared to controls (Fig. 2).

**Table 1:** Subject characteristics of RA patients and control in 2011

	RA (*n* = 177)	Control (*n* = 183)	*p* value	Effect size
Women	151 (85)	154 (84)	0.77	0.02
Age (years)	61.0 (18.0)	60.0 (16.0)	0.64	0.02
Height (cm)	155.8 (10.4)	157.0 (10.7)	0.012	0.13
Weight (kg)	54.0 (13.3)	54.8 (12.7)	0.18	0.07
BMI (kg/m^2^)	22.1 (4.8)	22.3 (4.2)	0.82	0.01
Smoking	51 (29)	26 (14)	0.001	0.18
Drinking habits	78 (44)	94 (51)	0.17	0.07
RF (IU/ml)	43 (98)	8 (3)	< 0.001	0.77
RF positive	134 (76)	20 (11)	< 0.001	0.65
Anti-CCP (U/ml)	67.6 (86.3)	0.6 (0.2)	< 0.001	0.81
Anti-CCP positive	152 (86)	5 (3)	< 0.001	0.84
RA duration (years)	10.4 (15.5)	–	–	–
csDMARD user	162 (92)	–	–	–
bDMARD user	104 (59)	–	–	–
GC user	47 (27)	–	–	–
ESR (mm/h)	20 (25)	8 (8)	< 0.001	0.41
MMP-3 (ng/ml)	64.5 (66.6)	43.0 (23.3)	< 0.001	0.36
hs-CRP (mg/l)	0.11 (0.38)	0.03 (0.05)	< 0.001	0.44
mHAQ-DI	0.25 (0.88)	–	–	–
TJC	2 (5)	–	–	–
SJC	0 (2)	–	–	–
Patient VAS	28 (40)	–	–	–
DAS28-ESR	3.4 (2.1)	–	–	–

In age, height, weight, BMI, RF, anti-CCP, RA duration, ESR, MMP-3, hs-CRP, mHAQ-DI, TJC, SJC, patient VAS, and DAS28-ESR, data are shown as median (interquartile range) and other categorical data were shown as patient number (%). *bDMARDs* biological disease-modifying antirheumatic drugs, *BMI* body mass index, *CCP* anti-cyclic citrullinated peptide antibody, *csDMARDs* conventional synthetic disease-modifying antirheumatic drugs, *DAS28-ESR* disease activity scores in 28 joints using erythrocyte sedimentation rates, *E-DII* energy-adjusted dietary inflammatory index, *GC* glucocorticoid, *hs-CRP* high-sensitivity C-reactive protein, *mHAQ-DI* modified health assessment questionnaire disability index, *MMP* matrix metalloproteinase, *RA* rheumatoid arthritis, *RF* rheumatoid factor, *SJC* swollen joint count, *TJC* tender joint count, *VAS* visual analog scale

### The association between E-DII scores and age

Because E-DII score were lower in 2017 compared to 2011 in both RA patients and controls, to test the possibility that aging has an effect on E-DII, we examined the association between E-DII score and age in RA patients and controls. E-DII score showed a significant negative correlation with age in both RA patients and controls in 2011. A similar and stronger association was also confirmed in 2017 (Supporting Figure 1).

### Comparison of clinical parameters between the low and high E-DII score groups in RA patients

The basic characteristics of RA patients and controls according to E-DII (the E-DII score of zero or more was classified as E-DII positive group, pro-inflammatory, and E-DII score of less than zero was classified as E-DII negative group, anti-inflammatory) in 2011 are shown in Table 2 and Supporting Table 1, respectively. The results showed that the percentage of women, RA duration, ESR, and DAS28-ESR were significantly higher, and the number of patients who have a drinking habit was significantly lower in the E-DII score negative group than in the positive group. Although it was not statistically significant, age and percentage of non-smokers were higher in the E-DII score negative group than in the E-DII positive group.

**Table 2:** Subject characteristics in two groups according to E-DII score in 2011 in RA patients

	E-DII score negative (*n* = 113)	E-DII score positive (*n* = 64)	*p* value	Effect size
Women	103 (91)	48 (75)	0.007	0.22
Age (years)	62.0 (17.0)	59.5 (25.0)	0.13	0.09
Height (cm)	154.5 (9.9)	156.7 (13.5)	0.19	0.09
Weight (kg)	52.3 (13.4)	55.5 (15.1)	0.017	0.12
BMI (kg/m^2^)	22.0 (5.3)	22.9 (5.1)	0.06	0.10
Smoking	27 (24)	24 (38)	0.06	0.14
Drinking habits	42 (37)	36 (56)	0.018	0.19
RF (IU/ml)	46 (108)	40 (89)	0.49	0.06
RF positive	86 (76)	48 (75)	0.86	0.01
Anti-CCP (U/ml)	61.1 (86.2)	88.5 (89.9)	0.45	0.07
Anti-CCP positive	94 (83)	58 (91)	0.26	0.10
RA duration (years)	12.2 (17.4)	8.1 (12.5)	0.037	0.11
csDMARD user	102 (90)	60 (94)	0.58	0.06
bDMARD user	65 (58)	39 (61)	0.75	0.03
GC user	27 (24)	20 (31)	0.29	0.08
ESR (mm/h)	23 (27)	16 (25)	0.005	0.13
MMP-3 (ng/ml)	60.8 (63.0)	72.3 (93.8)	0.33	0.07
hs-CRP (mg/l)	0.13 (0.36)	0.09 (0.43)	0.72	0.05
mHAQ-DI	0.25 (0.81)	0.25 (0.97)	0.43	0.07
TJC	2 (6)	1 (3)	0.06	0.10
SJC	0 (2)	0 (2)	0.44	0.07
Patient VAS	30 (40)	24 (40)	0.60	0.05
DAS28-ESR	3.6 (2.2)	3.2 (1.7)	0.007	0.12

In Age, Height, Weight, BMI, RF, Anti-CCP, RA duration, ESR, MMP-3, hs-CRP, mHAQ-DI, TJC, SJC, Patient VAS, DAS28-ESR, data were showed as median (interquartile range) and other categorical data were shown as patient number (%). There was no patient showed E-DII score zero. *bDMARDs* biological disease-modifying antirheumatic drugs, *BMI* body mass index, *CCP* anti-cyclic citrullinated peptide antibody, *csDMARDs* conventional synthetic disease-modifying antirheumatic drugs, *DAS28-ESR* disease activity scores in 28 joints using erythrocyte sedimentation rates, *E-DII* energy-adjusted dietary inflammatory index, *GC* glucocorticoid, *hs-CRP* high-sensitivity C-reactive protein, *mHAQ-DI* modified health assessment questionnaire disability index, *MMP* matrix metalloproteinase, *RA* rheumatoid arthritis, *RF* rheumatoid factor, *SJC* swollen joint count, *TJC* tender joint count, *VAS* visual analog scale

### Association between DII and disease activity in RA patients

**Fig. 3:**
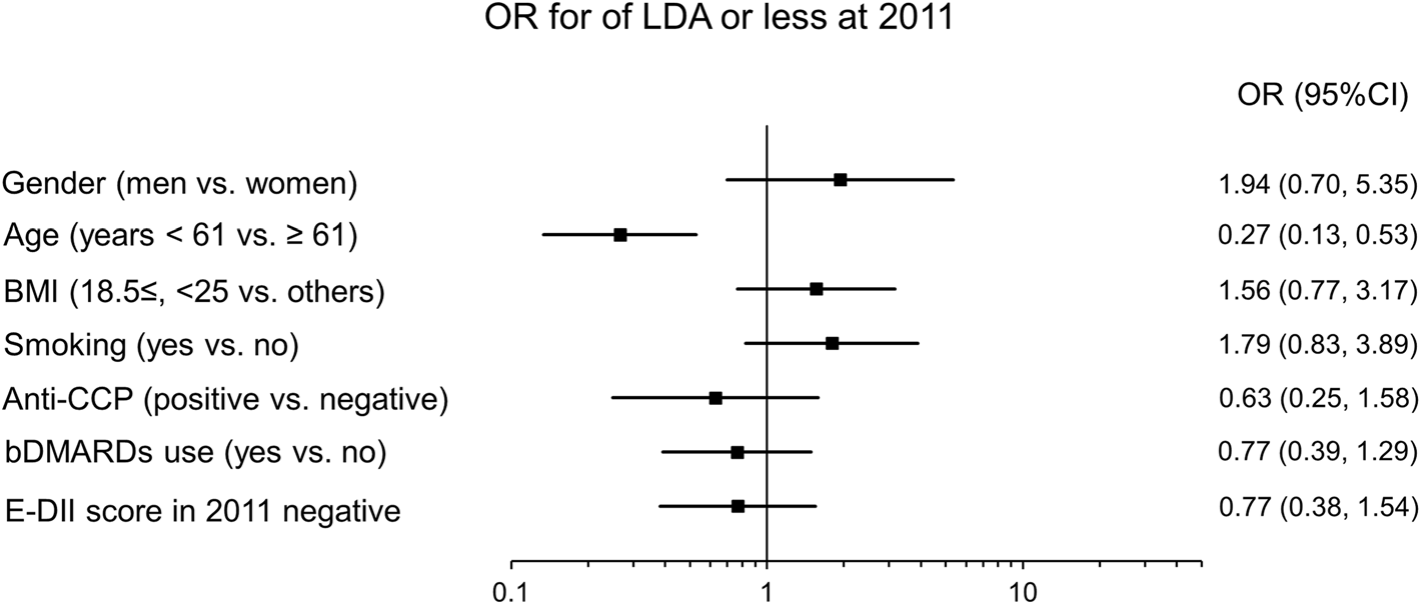
The effect of E-DII in 2011 on LDA or less at 2011. Data were analyzed with multivariable logistic regression analysis, and results were shown as forest plot of the odds ratio for low disease activity (LDA; DAS28-ESR ≤ 3.2) or less at 2011. The forced-entry method was used for the seven covariates shown in the figure. E-DII score was divided by positive or negative (i.e., positive as referent). bDMARDs, biological disease-modifying antirheumatic drugs; BMI, body mass index; CCP, cyclic citrullinated peptide antibody; CI, confidence interval; DAS28-ESR, disease activity score with 28 joint using erythrocyte sedimentation rate; E-DII, energy-adjusted dietary inflammatory index; OR, odds ratio

**Fig. 4:**
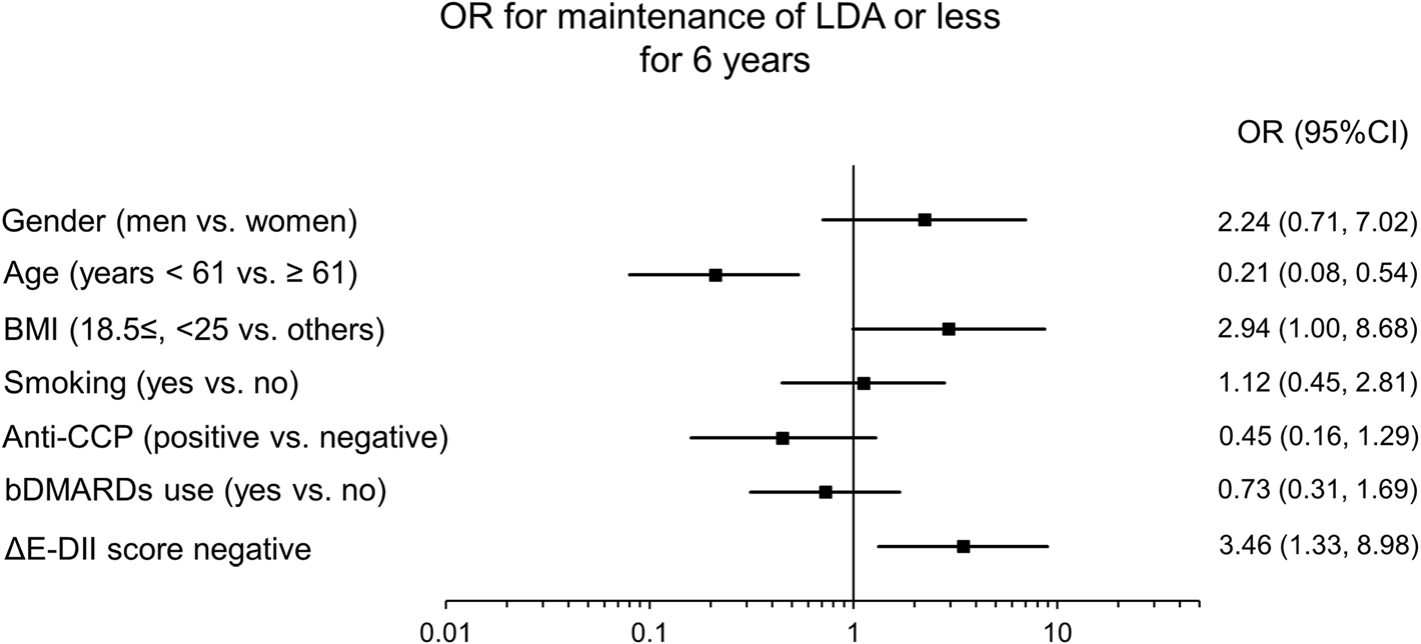
The effect of changes in E-DII on maintenance of LDA or less over 6-year period. Data were analyzed with multivariable logistic regression analysis, and results were shown as forest plot of the odds ratio for maintenance low disease activity (LDA; DAS28-ESR ≤ 3.2) or less for a 6-year period from 2011 to 2017. ΔE-DII indicates the change of the score from 2011 to 2017. The forced-entry method was used for the seven covariates shown in the figure. ΔE-DII score was divided by change value positive or negative (i.e., positive as referent). bDMARDs, biological disease-modifying antirheumatic drugs; BMI, body mass index; CCP, cyclic citrullinated peptide antibody; CI, confidence interval; DAS28-ESR, disease activity score with 28 joint using erythrocyte sedimentation rate; E-DII, energy-adjusted dietary inflammatory index; OR, odds ratio

The DAS28-ESR was higher in the E-DII score negative group than the positive group. Because factors such as gender and age also may affect disease activity, we examined the association between E-DII score and DAS28-ESR in detail. We classified RA patients as DAS28-ESR 3.2 or less or higher than 3.2, and the association between positive and negative of E-DII score adjusted by basic patient characteristics (gender, age, BMI, bDMARD use, smoking, and anti-CCP antibody positivity) was examined by multivariable logistic regression analysis. A cross-sectional analysis conducted using 2011 data showed no significant association between DAS28-ESR and E-DII score (Fig. 3, Supporting Table 2). We also examined longitudinally the association of change in E-DII score and RA patients who had been able to maintain LDA or less for 6 years from 2011 to 2017, and adjusted for same covariates in 2011 cross-sectional analysis. The analysis focused on the anti-inflammatory change in E-DII score, from 2011 to 2017 (ΔE-DII score negative). Anti-inflammatory change in E-DII score (lower E-DII score in 2017 compared to 2011) was a significant factor (OR 3.46, 95% CI 1.33–8.98, *p* = 0.011) associated with LDA for 6 years (Fig. 4, Supporting Table 2). In the analysis of another model, the presence or absence of drinking habits was a non-significantly associated factor (Supporting Table 3).

The degree of disease activity improvement from 2011 to 2017 was assessed according to the EULAR response, and the association of E-DII score in the 54 subjects with changes according to the EULAR response was examined using multivariable logistic regression analysis. Anti-inflammatory change in E-DII score (OR 0.95; 95% CI 0.47–1.91, *p* = 0.88) showed no significance.

## Discussion

In the present study, we used the E-DII, which quantifies the inflammatory potential of diet based on reported intake and controlling for energy density of the diet. We investigated E-DII characteristics and their association with disease activity in RA patients, including comparison with non-RA controls. The results suggested that E-DII was higher (pro-inflammatory) in RA patients than in controls, and that the daily nutrient composition based on reported dietary intake and were more likely to trigger inflammation in RA patients. The results were the same at two separate time points, in 2011 and 2017. These results indicate that diet-associated inflammation may be related to the pathogenesis of RA. The association between E-DII and the onset of RA needs to be investigated in large prospective cohort studies of non-RA subjects to determine whether E-DII had any effect on the development of newly incident RA. A large prospective cohort study has examined the association between Empirical Dietary Inflammatory Pattern (EDIP) calculated from 18 foods and beverages and the development of RA. The results suggest that EDIP may be associated with the development of RA, albeit limited to subsets of both age and gender [28]. However, EDIP is based on reduced rank regression of food items commonly consumed in the USA and hence cannot be generalized to all populations. In addition, the Alternative Healthy Eating Index (AHEI), which assesses dietary quality, has also been reported to be associated with the onset of RA [29]. Similar prospective studies using the E-DII will be needed in the future.

Our data showed that E-DII score was negatively correlated with age with reproducibility, and diets of older adults were more anti-inflammatory dietary. In addition, E-DII was lower in 2017 compared to 2011, not only in RA but also in controls. These results suggest that aging may influence DII scores in the presence or absence of RA. The inverse relationship between age and DII score has been seen in other [30, 31], but not all populations [32–35]. A survey of Japanese residents aged 55 years and older reported that the proportion of people who subjectively perceive their health to be poor increases with age, and the proportion of people who try to eat a well-balanced diet also increases with age [36]. This may have been one of the factors that influenced the association between age and E-DII.

In our analysis, there were significantly higher numbers of males in the E-DII positive group compared to the negative group. In the abovementioned research in Japan, men were nearly 5–20% less likely than women to take care of their diet for their health [36]; this also depends on age, and this may be a factor that helps to explain our result.

Because of the higher E-DII score in RA patients compared to controls, we examined the association of E-DII with parameters such as disease activity in RA by comparing the E-DII score positive group and the negative group. The E-DII score negative group had a higher DAS28-ESR; however, when multiple basic characteristics of the patient are adjusted, there was no significant association between DAS28-ESR and E-DII score. Drinking habits themselves were also not associated with disease activity.

However, in the longitudinal analysis, anti-inflammatory change in E-DII score was associated with maintaining LDA for 6 years. On the other hand, there was no significant association between change in disease activity such as of EULAR response level, and E-DII. This result might indicate the impact of E-DII on controlling disease activity may be less important and may contribute to maintaining disease activity in patients whose base disease activity is relatively well maintained. However, there are currently few reports of control of long-term disease activity and changes in diet and nutrient intake in patients with RA. However, because this was an observational study that did not involve dietary counseling, the relationship between changes in E-DII scores and disease activity needs to be verified in intervention studies. In fact, a randomized controlled trial (RCT) has been reported that verified that switching dietary content to a pattern that is expected to have anti-inflammatory effects suppressed disease activity after 10 weeks [11]. Thus, more long-term disease intervention studies are warranted.

Although it has been reported that a vegetarian diet, i.e., excluding animal products, improves disease activity in RA patients, there are issues that need to be resolved, such as unintended weight loss due to reduced energy intake and difficulty in adhering to the diet [37]. Although the number of dropouts due to difficulties in adherence to the diet is not described in the crossover RCT dietary intervention described above, it is not necessary to completely restrict animal products; thus, the anti-inflammatory diet may not appear drastically different from ones normal daily diet [11] and may be relatively easy to continue. In the diet of patients with RA, it is important to note that there are few side effects and high compliance with the diet in the subjects, therefore providing an ideal situation in which to validate long-term effects, and dietary interventions focusing on anti-inflammatory effects.

Despite its strengths as a prospective cohort, the limitations of this study need to be considered. First, this study was conducted at one institution and the number of subjects was relatively small. Only a small number of patients had their RA under control for 6 years with LDA or less (*n* = 35, 19.8%). Therefore, a prospective multicenter, cohort study with a large number of patients is desirable in order to increase statistical power. Second, there were nearly four times as many women as men in this study. Although epidemiologic evidence indicates that women have a higher prevalence of RA, research studies that focus on men also are needed. Third, the E-DII score is calculated from self-reported intake. So, there are limitations in both the nature of self-report and the nutrient database on which the values are calculated. Fourth, this study suggests that E-DII may affect the maintenance of disease activity; studies also are needed to examine the relationship between the intake of each nutrient or food alone, which is necessary for the calculation of E-DII, and disease activity in RA patients.

## Conclusions

The diets of RA patients had a higher inflammatory potential than controls. Although E-DII score was not a factor associated with significant disease activity change, anti-inflammatory change in E-DII score appeared to be associated with maintaining low disease activity in patients with RA.

## Supplementary Information

**Additional file 1 Supporting Figure 1** Scatter plots of E-DII and age in both control and RA patient groups. A: E-DII and age (years) in controls in 2011. B: E-DII and age (years) in RA patients in 2011. C: E-DII and age (years) in controls in 2017. D: E-DII and age (years) in RA patients in 2017. The correlation coefficient was calculated as Spearman’s rank-order correlation coefficient. *E-DII* energy adjusted dietary inflammatory index, *RA* rheumatoid arthritis.

**Additional file 2.**

## Data Availability

The datasets used and/or analyzed during the current study are available from the corresponding author on reasonable request.

## Abbreviations

ADL: Activity of daily living
BDHQ: Brief-type self-administered diet history questionnaire
bDMARDs: Biological disease-modifying antirheumatic drugs
BMI: Body mass index
CCP: Cyclic citrullinated peptide
CI: Confidence interval
csDMARDs: Conventional synthetic disease-modifying antirheumatic drugs
DAS28-ESR: Disease activity scores in 28 joints using erythrocyte sedimentation rates
E-DII: Energy-adjusted dietary inflammatory index
EULAR: European League against Rheumatism
GC: Glucocorticoid
hs-CRP: High-sensitivity C-reactive protein
JAK-STAT: Janus activated kinase-signal transducers and activators of transcription
LDA: Low disease activity
mHAQ-DI: Modified health assessment questionnaire disability index
MMP: Matrix metalloproteinase
OR: Odds ratio
PUFA: Polyunsaturated fatty acids
RA: Rheumatoid arthritis
RF: Rheumatoid factor
SJC: Swollen joint count
TJC: Tender joint count
TOMORROW: Total management of risk factors in rheumatoid arthritis patients to lower morbidity and mortality
tsDMARDs: Targeted synthetic disease-modifying antirheumatic drugs
VAS: Visual analog scale
