# Supplementary figures and images for: Change in dietary inflammatory index score is associated with control of long-term rheumatoid arthritis disease activity in a Japanese cohort: the TOMORROW study
Source: Arthritis Res Ther. 2021 Apr 8;23:105. doi: 10.1186/s13075-021-02478-y (PMC8028141; doi:10.1186/s13075-021-02478-y)

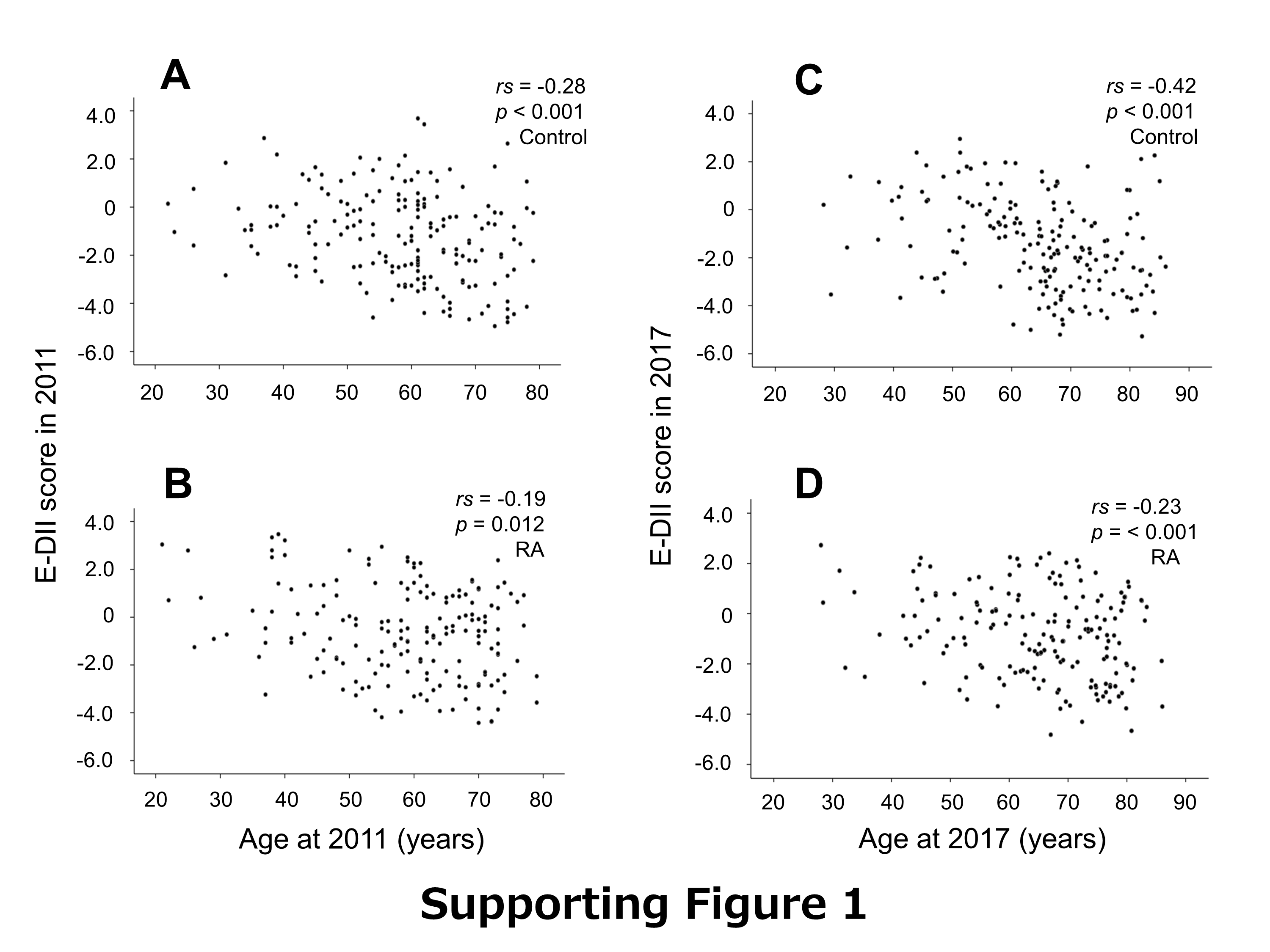

Supplement: Supplementary file 1 — Additional file 1 Supporting Figure 1 Scatter plots of E-DII and age in both control and RA patient groups. A: E-DII and age (years) in controls in 2011. B: E-DII and age (years) in RA patients in 2011. C: E-DII and age (years) in controls in 2017. D: E-DII and age (years) in RA patients in 2017. The correlation coefficient was calculated as Spearman’s rank-order correlation coefficient. E-DII energy adjusted dietary inflammatory index, RA rheumatoid arthritis. [file 13075_2021_2478_MOESM1_ESM.tif]
