# Supplementary material for: Change in dietary inflammatory index score is associated with control of long-term rheumatoid arthritis disease activity in a Japanese cohort: the TOMORROW study
Source: Arthritis Res Ther. 2021 Apr 8;23:105. doi: 10.1186/s13075-021-02478-y (PMC8028141; doi:10.1186/s13075-021-02478-y)
Supplement: Supplementary file 2 — Additional file 2. [file 13075_2021_2478_MOESM2_ESM.docx]

| Supporting Table1 Subject characteristics in two groups according to E-DII score in 2011 in Control | | | | |  |
| --- | --- | --- | --- | --- | --- |
|  | E-DII score  negative (n = 132) | E-DII score  positive (n = 51) | p value | effect size |  |
|  |  |  |  |  |  |
| Women | 14 (11) | 15 (29) | 0.003 | 0.23 |  |
| Age (years) | 61.0 (17.0) | 58.0 (16.0) | 0.005 | 0.21 |  |
| Height (cm) | 155.1 (9.3) | 161.3 (10.6) | <0.001 | 0.27 |  |
| Weight (kg) | 54.0 (11.3) | 58.7 (14.2) | 0.03 | 0.16 |  |
| BMI (kg / m^2^) | 22.2 (4.1) | 22.7 (4.6) | 0.45 | 0.06 |  |
| Smoking | 16 (12) | 10 (20) | 0.24 | 0.10 |  |
| Drinking habits | 61 (46) | 33 (65) | 0.032 | 0.17 |  |
| RF (IU / ml) | 3 (0) | 3 (1) | 0.34 | 0.07 |  |
| RF positive | 13 (10) | 7 (14) | 0.44 | 0.06 |  |
| Anti-CCP (U / ml) | 0.6 (0.1) | 0.6 (0.3) | 0.26 | 0.08 |  |
| Anti-CCP positive | 4 (3) | 1 (2) | 1.00 | 0.03 |  |
| ESR (mm / hr) | 9 (10) | 8 (8) | 0.12 | 0.11 |  |
| MMP-3 (ng / ml) | 41.8 (23.1) | 46.1 (25.2) | 0.38 | 0.07 |  |
| hs-CRP (mg / l) | 0.03 (0.04) | 0.03 (0.05) | 0.80 | 0.02 |  |
| In Age, Height, Weight, BMI, RF, CCP, ESR, MMP-3, hs-CRP, data were showed as median (inter quarter range) and other categorical data were shown as patient number (%). There was no subjects showed E-DII score zero. BMI body mass index, CCP cyclic citrullinated peptide antibody, E-DII energy adjusted dietary inflammatory index, hs-CRP high sensitivity C-reactive protein, MMP matrix metalloproteinase, RF rheumatoid factor | | | | |  |
|  |  |  |  |  |  |
|  |  |  |  |  |  |
|  |  |  |  |  |  |

| Supporting Table 2 Bootstrapping confidence interval for each analysis | | |  |
| --- | --- | --- | --- |
|  | items | 95% CI |  |
| For Figure 3 | E-DII score in 2011 negative | 0.35, 1.76 |  |
| For Figure 4 | ΔE-DII score negative | 1.14, 8.76 |  |
| Bootstrap confidence intervals are calculated to ensure confidence intervals (repeated 1000 times). E-DII; energy adjusted dietary inflammatory index, CI; confidence interval | | |  |
|  |  |  |  |
|  |  |  |  |

| Supporting Table 3 The effect of drinking habits on maintenance of LDA or less over 6-year period | |
| --- | --- |
|  | OR (95% CI) |
| Gender | 2.29 (0.74, 7.08) |
| Age | 0.23 (0.09, 0.61) |
| BMI | 3.48 (1.21, 10.00) |
| Smoking | 1.00 (0.40, 2.48) |
| Anti-CCP | 0.51 (0.18, 1.46) |
| bDMARDs use | 0.87 (0.38, 1.97) |
| Drinking habits | 1.68 (0.71, 3.97) |
| Data were analyzed with multivariable logistic regression analysis, and results were shown as the odds ratio for maintenance low disease activity (LDA; DAS28-ESR≤3.2) or less for a 6-year period from 2011 to 2017. The forced-entry method was used for the seven covariates shown in the table. The odds for gender was shown as odds for men (i.e., women as referent), the odds for age was shown as odds for years ≥ 61 (i.e., years < 61 as referent), the odds for BMI was shown as odds for standard (18.5≤, <25) (i.e., BMI < 18.5, and ≥25 as referent), the odds for anti-CCP was shown as odds for positive (i.e., negative as referent), the odds for bDMARDs use, smoking, drinking habits were shown as odds for yes (i.e., no as referent). bDMARDs biological disease-modifying antirheumatic drugs, CCP; cyclic citrullinated peptide antibody, CI; confidence interval, DAS28-ESR; disease activity score with 28 joint using erythrocyte sedimentation rate, E-DII; energy-adjusted dietary inflammatory index, LDA; low disease activity, OR; odds ratio | |
|  |  |
|  |  |
|  |  |
|  |  |
|  |  |
|  |  |
|  |  |
